# Blood and brain transcriptome analysis reveals *APOE* genotype-mediated and immune-related pathways involved in Alzheimer disease

**DOI:** 10.1186/s13195-022-00975-z

**Published:** 2022-02-09

**Authors:** Rebecca Panitch, Junming Hu, Weiming Xia, David A. Bennett, Thor D. Stein, Lindsay A. Farrer, Gyungah R. Jun

**Affiliations:** 1 Department of Medicine (Biomedical Genetics), Boston University School of Medicine, 72 East Concord Street, Boston, MA 02118 USA; 2 Department of Pharmacology & Experimental Therapeutics, Boston University School of Medicine, 72 East Concord Street, Boston, MA 02118 USA; 3 Department of Veterans Affairs Medical Center, Bedford, MA 01730 USA; 4 Rush Alzheimer’s Disease Center, Rush University Medical Center, 1750 W. Harrison Street, Suite 1000, Chicago, IL 60612 USA; 5 Department of Pathology & Laboratory Medicine, Boston University School of Medicine, 72 East Concord Street, Boston, MA 02118 USA; 6 Department of Neurology, Boston University School of Medicine, 72 East Concord Street, Boston, MA 02118 USA; 7 Department of Ophthalmology, Boston University School of Medicine, 72 East Concord Street, Boston, MA 02118 USA; 8 Department of Biostatistics, Boston University School of Public Health, 715 Albany Street, Boston, MA 02118 USA; 9 Department of Epidemiology, Boston University School of Public Health, 715 Albany Street, Boston, MA 02118 USA

**Keywords:** Alzheimer’s disease, Blood-brain barrier, *APOE*, Differential expression, Co-expression network, Vascular injury

## Abstract

**Background:**

While Alzheimer disease (AD) is generally considered as a brain disorder, blood biomarkers may be useful for the diagnosis and prediction of AD brain pathology. The *APOE* ε4 allele has shown cerebrovascular effects including acceleration of blood-brain barrier (BBB) breakdown.

**Methods:**

We evaluated the differential expression of previously established AD genes in brains from 344 pathologically confirmed AD cases and 232 controls and in blood from 112 pathologically confirmed AD cases and 67 controls from the Religious Orders Study and Memory and Aging Project. Differential gene expression between AD cases and controls was analyzed in the blood and brain jointly using a multivariate approach in the total sample and within *APOE* genotype groups. Gene set enrichment analysis was performed within *APOE* genotype groups using the results from the combined blood and brain analyses to identify biologically important pathways. Gene co-expression networks in brain and blood samples were investigated using weighted correlation network analysis. Top-ranked genes from networks and pathways were further evaluated with vascular injury traits.

**Results:**

We observed differentially expressed genes with *P* < 0.05 in both brain and blood for established AD genes *INPP5D* (upregulated) and *HLA-DQA1* (downregulated). *PIGHP1* and *FRAS1* were differentially expressed at the transcriptome-wide level (*P* < 3.3 × 10^−6^) within ε2/ε3 and ε3/ε4 groups, respectively. Gene set enrichment analysis revealed 21 significant pathways (false discovery rate *P* < 0.05) in at least one *APOE* genotype group. Ten pathways were significantly enriched in the ε3/ε4 group, and six of these were unique to these subjects. Four pathways (allograft rejection, interferon gamma response, peroxisome, and TNFA signaling via NFKB) were enriched for AD upregulated genes in the ε3/ε4 group and AD downregulated genes in subjects lacking ε4. We identified a co-expressed gene network in the brain that reproduced in blood and showed higher average expression in ε4 carriers. Twenty-three genes from pathway and network analyses were significantly associated with at least one vascular injury trait.

**Conclusion:**

These results suggest that the *APOE* genotype contributes to unique expression network profiles in both blood and brain. Several genes in these networks are associated with measures of vascular injury and potentially contribute to ε4’s effect on the BBB.

**Supplementary Information:**

The online version contains supplementary material available at 10.1186/s13195-022-00975-z.

## Background

Alzheimer disease (AD) is a neurodegenerative disorder characterized by amyloid plaques and neurofibrillary tau tangles in the brain [1]. Because these hallmark proteins are sometimes detectable in blood before clinical symptoms appear, there are on-going efforts to identify blood-based signatures from multi-omics and biomarker data that can facilitate detection of AD preclinically [2, 3]. For example, plasma phosphorylated tau levels are highly correlated with neurodegenerative disorders and AD pathology [4, 5].

Cerebrovascular AD-related pathology that may affect the blood-brain barrier (BBB), such as cerebral amyloid angiopathy (CAA), has been shown to exacerbate neurodegeneration and neuroinflammation [6]. Dysfunction of the BBB, a semi-permeable border separating the extracellular fluid and brain tissue from circulating blood, has been implicated in the accumulation of amyloid-β (Aβ) and hyperphosphorylation of tau protein [7, 8]. Apolipoprotein E (*APOE*) genotype is the strongest genetic risk factor for late-onset AD and the ε4 allele has been recently associated with BBB dysfunction leading to cognitive decline [9, 10]. Heterozygosity of the *APOE* ε4 allele confers a 3–4-fold increase of AD risk and ε4 homozygotes have a 10–12-fold increased likelihood of a clinical diagnosis of AD among persons of European ancestry [9, 11]. By contrast, among clinically and neuropathologically confirmed AD cases and controls of European ancestry, a single copy of the *APOE* ε2 allele is associated with 0.61-fold decreased risk and ε2 homozygotes have an 0.87-fold reduced risk for AD compared to individuals with the ε3/ε3 genotype [12]. Cerebrovascular AD-related pathologies have also shown *APOE* genotype-dependent patterns. Both ε2 and ε4 are significantly associated with the risk of CAA [13].

Previous whole transcriptome-wide studies from autopsied brains demonstrate that the classical complement cascade and tau phosphorylation are linked to AD in an *APOE* genotype-specific manner [14, 15]. However, expression profiles associated with AD have not been intensively investigated in the blood and brain from the same individuals, especially separated by the *APOE* genotype. Here, we analyzed gene expression measured in the blood and brain tissue obtained from participants of the Religious Orders Study and Rush Memory and Aging Project (ROSMAP) [16] stratified by the *APOE* genotype in order to discern AD-related differential gene expression, biological pathways, and gene networks shared in the blood and brain.

## Methods

### Sources of blood transcriptomic and phenotypic data

RNA-sequencing (RNA-seq) data generated from blood donated by 614 ROSMAP participants and phenotypic data collected from those subjects were obtained from the Synapse portal [17]. RNA batches were prepared using a SMART-seq2 protocol (batches 1-2) or a SMART-seq2-like protocol (batch 3). Batch 1 containing 47 samples (2 × 101bp) and batch 2 containing 201 samples (2 × 76bp) were pooled and sequenced by HiSeq 2500 (Illumina). Batch 3 containing 366 samples (2 × 50 bp) was pooled and sequenced on Nova Seq 6000 (Illumina) (Supplementary Table 1). A post-mortem diagnosis of AD was established for 112 participants using NIA-Reagan criteria including Braak staging for assessing the severity of neurofibrillary tangles and the Consortium to Establish a Registry for Alzheimer Disease (CERAD) semi-quantitative measure for neuritic plaques (CERAD score). Another 67 participants who were clinically normal showed no pathological evidence of AD and were included in this study as controls (Table 1) [16, 18]. Age, sex, sequencing batch, and library batch information was available for all subjects.

**Table 1:** Number of ROSMAP participants with RNA-seq data by *APOE* genotype and batch

APOE genotype	Blood batch 1		Blood batch 2		Combined blood		Brain	Blood-brain overlap		
	AD	CTRL	AD	CTRL	AD	CTRL	AD	CTRL	AD	CTRL
ɛ2/ɛ2**	0	0	0	1	0	1	0	5	0	1
**ɛ2/ɛ3**	5	3	9	9	14	12	32	39	12	8
**ɛ3/ɛ3**	16	11	44	31	60	42	197	158	49	33
ɛ2/ɛ4**	1	1	5	0	6	1	9	4	4	1
ɛ3/ɛ4*	8	1	21	10	21	10	101	25	22	8
ɛ4/ɛ4**	1	0	1	0	0	0	5	1	2	0
NA	0	0	1	0	1	0	0	0	0	0
**Total**	31	16	81	51	112	67	344	232	89	51

Blood batch 3 was excluded because it contained controls only

*APOE ɛ3/ɛ4 subjects from blood batch 1 were excluded from all analyses due to the small sample size

**ɛ2/ɛ2, ɛ2/ɛ4, and ɛ4/ɛ4 subjects were not analyzed separately due to the small sample size

*NA*, *APOE* genotype not available

### Sources of brain transcriptomic and phenotypic data

Publicly available prefrontal cortex brain RNA-seq and neuropathological data for 639 ROSMAP participants were obtained from the Synapse portal [17] (Supplementary Table 1). Sequencing libraries were prepared using the strand-specific dUTP method with poly-A selection, and all samples were sequenced using an Illumina HiSeq instrument. Of these 639 samples, data from 576 samples with both RNA integrity number (RIN) and post-mortem interval (PMI) were included in subsequent differential expression analyses (Table 1). Samples with RIN < 5 were excluded from further study. Previously reported RNA-seq data were also available which were derived from the frontal cortex tissue region of 208 frontal autopsied brains (64 AD and 129 controls) donated to the Framingham Heart Study and Boston University Alzheimer’s Disease Center (FHS/ADRC) [19]. A diagnosis of AD in these brains was established using NIA-Regan criteria including Braak staging and CERAD score [19].

### Quality control, mapping, and quantification of gene expression data and sample

The 614 FASTQ files derived from blood RNA-seq data were processed in batches. Quality control (QC) of the sequence data was performed using FastQC which checked for overabundance of adaptors and overrepresented sequences [20]. Reads passing initial QC were aligned to the human reference genome (GRCh38.95) using STAR (version 2.6.1c), which implements 2-pass mapping to increase the chances of mapping splice reads from novel junctions [21, 22]. To account for differences in read lengths between batches, we created three genomic alignment index files with read lengths of 50bp, 76bp, and 101bp, respectively, for mapping the study samples to the reference genome.

The 639 binary alignment map (BAM) files containing brain RNA-seq data required additional processing before alignment and thus were converted to FASTQ files using the FastqTosam function in Picard tools [23]. Samples were checked for adaptor overabundance and overrepresented sequences using FastQC [20]. Paired-end reads were aligned to the human reference genome as described above. In order to map brain samples to the reference genome, genomic index files (read length = 101bp) were created.

The resulting BAM files for each brain and blood sample contained mapped paired-end reads and a corresponding alignment report file. Gene and isoform levels were quantified using RSEM (version 1.3.1) [24] and Bowtie2 (version 2.3.4.1) [25] and then annotated using *Homo sapiens* GRCh38.95.gtf annotation files. Files generated by this process for each sample contained several variables for each gene including gene id, gene length, effective gene length, expected count, counts per million (CPM), and fragments per kilobase of exon model per million reads mapped (FPKM) reads.

### Gene expression analysis in the blood and brain

#### Differential expression analysis

Genes with less than two reads on average among 80% or more of the samples were excluded from analyses. Blood and brain samples were corrected for between-sample variability using a trimmed mean of *M*-value normalization method [26]. Differential gene expression analysis between AD and control subjects in the blood and brain was performed separately using the VOOM and LIMMA software [27, 28]. For differential gene expression analysis in the brain, the normalized expression of each gene was compared between AD cases and controls using linear regression models adjusting for sex, age at death, RNA integrity number (RIN), post-mortem interval (PMI), and sequencing batch as covariates. Gene expression analysis of the blood samples included only the 179 individuals who were neuropathologically examined and models included covariates for sex, age at exam, and library batch. A total of 140 individuals had genomic data derived from the blood and brain and included in analyses for both tissues. Analyses were performed in the total sample and subgroups defined by the *APOE* genotype (ε2/ε3, ε3/ε3, and ε3/ε4). Subjects with genotypes ε2/ε2, ε2/ε4, and ε4/ε4 were excluded from analyses due to small samples sizes (Supplementary Table 1, Table 1). Analyses of gene expression in blood were further stratified by RNA batch due to differences in read length and sample substructure (Supplementary Fig. 1a), while we did not observe batch differences in the brain (Supplementary Fig. 1b). For the ε3/ε4 subgroup, data from batch 2 in blood were only analyzed because the batch 1 sample size was too small (Table 1). Analyses were not stratified by batch in the brain because there was no obvious batch effect and sample sizes in each of the nine batches were too small, especially within the ε2/ε3 and ε3/ε4 subgroups (Supplementary Table 2). Results from analyses of each batch and *APOE* genotype group were combined by meta-analysis weighting for the number of AD cases and accounting for effect direction using the METAL program [29].

To evaluate differential gene expression patterns in the joint blood and brain datasets, we combined univariate results from the blood and brain using the R package CUMP which incorporates O’Brien’s method [30]. In this method, a combined *Z*-score was calculated using *t*-value estimates derived from the LIMMA linear regression analyses and/or from *Z*-score estimates from the meta-analysis of the blood batches. All analyses were weighted by the number of samples within *APOE* genotype groups or in the total sample.

#### Single-cell gene expression analysis

A normalized single-cell RNA-sequencing expression matrix from ~2400 cells collected from the blood of healthy individuals ages 25 to 40 and proportions of dendritic cells and monocytes for each sample were obtained from the Single Cell Portal [31]. Additional details of these subjects and single-cell RNA sequencing are reported elsewhere [32]. FASTQ single nuclei RNA-sequencing data from the prefrontal cortex of 48 brains from ROSMAP participants (24 AD cases, 24 controls) were obtained from the Synapse portal [17] and processed as previously described [19]. Among the 48 individuals in this dataset, 26 are included in the ROSMAP bulk brain RNA-seq dataset and four of these 48 individuals overlap with the ROSMAP bulk blood RNA-seq dataset. The average expression for each cell type in the blood and brain RNA-seq datasets was calculated for each gene.

#### Gene set enrichment analysis

Differentially expressed genes in the total sample or within *APOE* genotype groups were ranked by a combined *Z*-score from the blood and brain using the O’Brien method. Gene set enrichment analysis was performed using this ranked list and hallmark gene set pathway information obtained from the Molecular Signatures Database (MSigDB) as previously described [33, 34]. The hallmark gene set is focused on biological processes obtained by aggregated MsigDB signatures. Pathway enrichment scores were determined based on the degree to which a set of genes was overrepresented by the largest positive and smallest negative *Z*-scores. Genes that contributed the most to the enrichment score of each pathway were designated as leading-edge genes.

### Co-expressed gene network analysis in the blood and brain

Co-expressed genes in networks were identified using 14,456 coding genes in the brain and 11,379 coding genes in the blood in the ROSMAP RNA-seq dataset using the weighted gene correlation network analysis (WGCNA) algorithm [35]. Analyses of data from blood included only 141 batch 2 samples with and without post-mortem examination to avoid batch effects, and analyses of data from the brain comprised 636 samples excluding lack of RIN or batch information (Supplementary Table 3). We used gene expression levels calculated as log-transformed fragments per kilobase of transcript per million (FPKM). Soft-power parameters of 12.0 and 12.5 were selected for analyses of brain and blood data, respectively, as previously described [19]. Expression data were clustered hierarchically by implementing a dissimilatory topological overlap matrix (TOM). Initial modules with a minimal network size of 100 genes were identified and labeled using dynamic tree cutting. Eigengenes were derived from the first principle component for each module and served as representative values of gene expression in a given module [36]. Networks with high eigengene similarity and a height of 0 were merged using the mergeCloseModules function in WGCNA. Fuzzy module membership was assigned using the signedKME function.

Network modules identified in the brain were examined for preservation in blood using the modulePreservation function in WGCNA. Brain networks with a Zsummary score > 5 were considered preserved in blood networks [37]. Relevance of the networks to AD pathology was established based on enrichment of AD-related genes that was determined using the userListEnrichment function in WGCNA. For the purpose of this analysis, we defined AD-related genes which included those within 20kb of single nucleotide polymorphisms (SNPs) showing at least modest evidence (*P* < 0.001) for association with AD risk [38] or AD-related neuropathological measures of Tau and Aβ proteins [39]. We used EnrichR to identify KEGG pathways enriched for AD-related genes in the preserved networks [40]. Next, genes in networks contributing to significant pathways were further evaluated using Ingenuity Pathway Analysis software (QIAGEN Inc.) to identify biological subnetworks.

### Measurements and association with vascular injury-related proteins

Intercellular adhesion molecule 1 (ICAM-1), vascular cell adhesion molecule 1 (VCAM-1), and serum amyloid α (SAA) were detected and measured in fresh tissue lysate from the dorsolateral prefrontal cortex area using the Mesoscale Discovery V-PLEX Plus Vascular Injury Panel Kit (Mesoscale Discovery, K15198G, Rockville MD). Gray matter was separated from frozen brain tissue on dry ice and weighed. Ice-cold RIPA buffer (ThermoScientific, #89901) was added to the gray matter at 5mL RIPA: 1g brain wet weight, and homogenized with Qiagen Tissue Lyser LT at 50Hz for 5 min (Qiagen, Germany) (ThermoScientific, Waltham MA). The homogenate was centrifuged at 17,000*g* at 4°C for 15 min, then the supernatant was aliquoted and stored at −80°C until use. Buffers and immunoassay plates were prepared according to the manufactory instructions and the brain homogenate was further diluted 5-fold. The immunoassay plates were read using the multi-detection SPECTOR 6000 Imager to quantitate protein levels (Mesoscale Discovery).

Additional analyses of 107 top-ranked genes emerging from pathways identified by differential gene expression and network analyses were performed using log-transformed FPKM values obtained previously from these FHS/ADRC donor brains [19]. Levels of ICAM-1, VCAM-1, and SAA proteins were rank-transformed after adjusting for age and sex. We performed association analyses using the expression levels of the selected genes with the levels of vascular injury-related proteins as quantitative outcomes in linear regression models further adjusting for RIN.

## Results

### Differentially expressed genes in the blood and brain

**Fig. 1:**
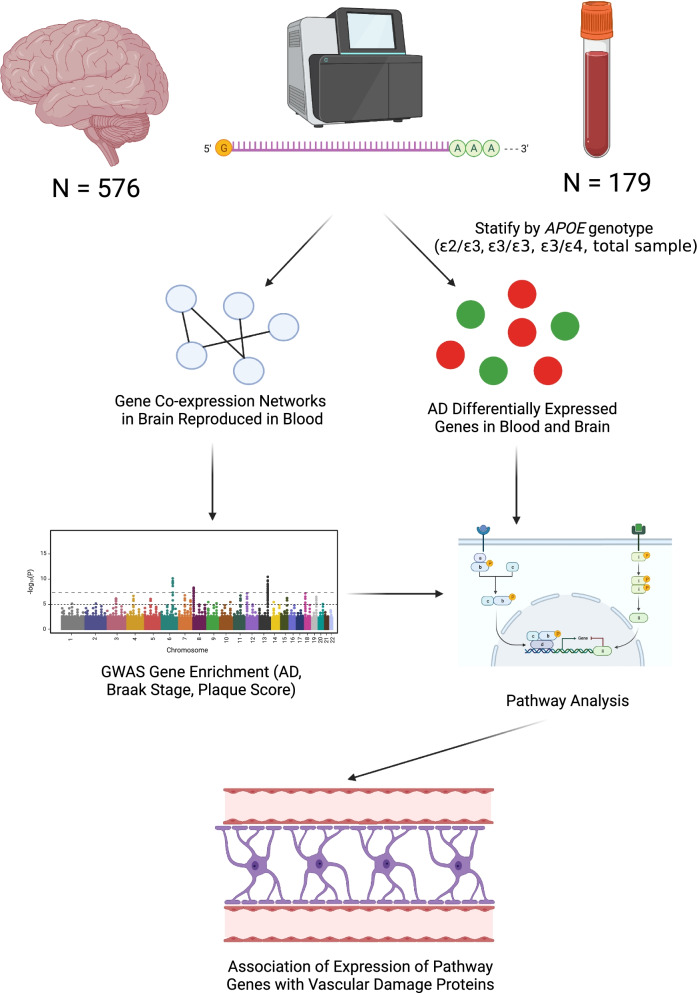
Analysis design and workflow. RNA-sequencing data were obtained from the blood and post-mortem frozen brain of neuropathologically verified AD cases and controls. Data were analyzed in two ways. First, gene co-expression analysis identified networks in the brain that reproduced in the blood. A second analysis identified genes differentially expressed between AD cases in controls in the total sample as well as within *APOE* genotype groups in both blood and brain. The expression of genes in the co-expression networks that were previously associated with AD by GWAS was tested for associated with AD-related traits measured in the brain. Next, genes in significant co-expression networks and differentially expressed genes in the blood and brain were incorporated as seeds in pathway analysis. Finally, the expression of genes from the most significant pathways was tested for association with levels of several vascular damage proteins. Figure created with BioRender.com

Gene expression levels in 179 blood and 576 brain samples from the ROSMAP dataset were compared between AD cases and controls (Fig. 1). In the total sample, no genes in the combined data from the blood and brain were differentially expressed at the transcriptome-wide significance level (*P* < 3.3 × 10^−6^). Of 78 genes containing or nearest to SNPs associated with AD at a genome-wide significance level in a recent large genome-wide association study (GWAS) [41], 64 passed QC and were expressed in both brain and blood. The expression of five of these 64 genes (*HLA-DQA1*, *INPP5D*, *SPDYE3*, *TSPOAP1*, and *SIGLEC11*) were nominally significant (*P* < 0.05) in the analysis of the combined blood and brain data (Table 2, Supplemental Table 4, Supplementary Fig. 2). Differential expression of *HLA-DQA1* and *INPP5D* was nominally significant at *P* < 0.05 in both blood and brain with the same direction of effect. Differentially expressed genes (DEGs) after multiple testing correction at *P* < 6.4 × 10^−4^ were evident only in the brain for *BCKDK* (*P* = 5.1 × 10^−4^, *P*_adj_ = 0.04), *TSPOAP1* (*P* = 2.6 × 10^−4^, *P*_adj_ = 0.02), and *SIGLEC11* (*P* = 1.6 × 10^−4^, *P*_adj_ = 0.01).

**Table 2:** Differentially expressed known AD genes in the combined blood and brain datasets in the total sample

Gene	Dataset	***APOE*** ɛ2/ɛ3			***APOE*** ɛ3/ɛ3			***APOE*** ɛ3/ɛ4			Total		
		***N***	***Z***-score	***P***-value	***N***	***Z***-score	***P***-value	***N***	***Z***-score	***P***-value	***N***	***Z***-score	***P***-value
INPP5D	Blood	26	−0.62	0.53	102	1.67	0.10	31	2.12	0.03	179	2.16	0.03
	Brain	71	1.80	0.07	355	0.92	0.36	126	0.66	0.51	576	2.30	0.02
	Combined	97	1.51	0.13	457	1.29	0.20	157	1.31	0.19	755	2.42	0.02
HLA-DQA1	Blood	26	−1.49	0.14	102	−1.80	0.07	31	0.96	0.34	179	−2.26	0.03
	Brain	71	0.30	0.76	355	−3.13	1.7 × 10^−3^	126	−1.01	0.31	576	−2.23	0.03
	Combined	97	−0.16	0.87	457	−2.84	4.6 × 10^−3^	157	−0.66	0.51	755	−2.42	0.02
SPDYE3	Blood	26	−0.08	0.94	102	−0.76	0.45	31	−0.96	0.34	179	−1.47	0.14
	Brain	71	−0.79	0.43	355	−0.97	0.33	126	−2.16	0.03	576	−2.73	6.4 × 10^−3^
	Combined	97	−0.77	0.44	457	−0.96	0.33	157	−2.37	0.02	755	−2.26	0.02
TSPOAP1	Blood	26	−1.38	0.17	102	0.30	0.77	31	−0.16	0.87	179	−0.55	0.58
	Brain	71	−1.04	0.30	355	−2.17	0.03	126	−1.85	0.06	576	−3.65	2.6 × 10^−4^
	Combined	97	−1.39	0.16	457	−1.35	0.18	157	−1.82	0.07	755	−2.24	0.03
SIGLEC11	Blood	26	1.43	0.15	102	0.47	0.64	31	0.24	0.81	179	0.33	0.74
	Brain	71	1.05	0.29	355	2.46	0.01	126	2.58	9.9 × 10^−3^	576	3.77	1.6 × 10^−4^
	Combined	97	1.42	0.16	457	1.86	0.06	157	2.54	0.01	755	2.17	0.03

The combined column reflects the meta-analysis results or single batch results in the case of one batch including low gene filtering

Two genes were differentially expressed between AD cases and controls at the transcriptome-wide level (*P* < 3.3 × 10^−6^) within a particular *APOE* genotype group. *PIGHP1* was significantly upregulated in AD cases in the combined brain and blood samples in the ε2/ε3 group (*Z* = 4.67, *P* = 3.1×10^−6^, *P*_adj_ = 0.05), a pattern predominated by the evidence in the brain but also apparent in the blood (Table 3, Supplementary Figs. 2, 3a). Among ε3/ε4 subjects, the expression of *FRAS1* was significantly downregulated in AD cases in blood only (*Z* = −4.66, *P* = 3.2 × 10^−6^, *P*_adj_ = 0.05) (Supplementary Fig. 3b). No transcriptome-wide significant DEGs were identified in the brain from any *APOE* genotype groups. Among genes previously associated with AD among ε2/ε3 subjects [19], *C4A*, *C4B*, and *HSPA2* were moderately (*P* < 10^−3^) upregulated in the brain but not blood from AD ROSMAP Study participants in the ε2/ε3 subgroup and total sample (Supplemental Table 5). Notably, *C4B* expression trended in the opposite direction (i.e., downregulated in AD cases) in blood from ε2/ε3 subjects (*P* = 0.08, *P*_adj_ = 1.0).

**Table 3:** Novel differentially expressed genes in the blood or brain within *APOE* genotype groups

Gene	Dataset	***APOE*** ɛ2/ɛ3			***APOE*** ɛ3/ɛ3			***APOE*** ɛ3/ɛ4			Total		
		***N***	***Z***-score	***P***-value	***N***	***Z***-score	***P***-value	***N***	***Z***-score	***P***-value	***N***	***Z***-score	***P***-value
FRAS1	Blood	NA	NA	NA	75	−0.24	0.81	31	−4.66	**3.2 × 10**^**−6**^	132	−2.26	0.02
	Brain	NA	NA	NA	355	−0.97	0.33	126	0.28	0.78	576	−0.49	0.62
	Combined	NA	NA	NA	430	−0.75	0.45	157	−1.23	0.22	708	−1.27	0.20
PIGHP1	Blood	26	1.67	0.09	102	0.43	0.67	31	1.30	0.19	179	1.14	0.25
	Brain	71	4.42	9.8 × 10^−6^	355	0.53	0.59	126	0.47	0.64	576	1.98	0.05
	Combined	97	4.67	**3.1 × 10**^−**6**^	457	0.53	0.59	157	0.87	0.39	755	1.68	0.09

*NA* not available due to low expression. Bolded *P*-values pass transcriptome-wide multiple testing threshold (3.3 × 10^−6^)

Examination of cell-level expression profiles of the DEGs in Tables 1 and 2 revealed that in blood cell types *HLA-DQA1* and *INPP5D* were more highly expressed in dendritic cells and monocytes compared to other genes in this group (Supplementary Fig. 4a). *INPP5D* was the only gene in this group expressed in brain cell types and specifically in microglia (Supplementary Fig. 4b).

### APOE genotype-dependent pathways in combined blood and brain expression profiles

**Fig. 2:**
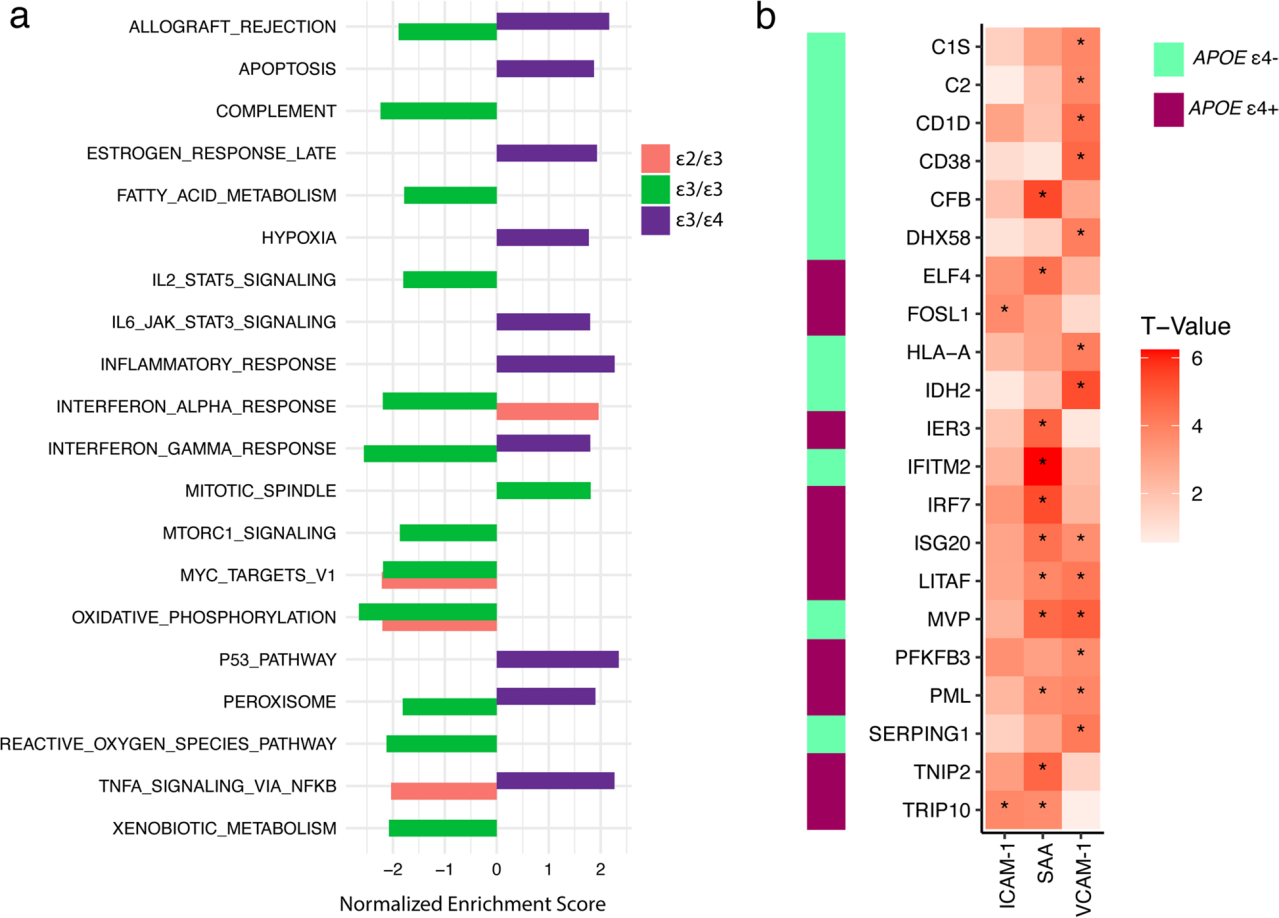
Significant pathways in the blood and brain by the *APOE* genotype. **a** Significant pathways (adjusted *P* < 0.05) within *APOE* genotype groups that are enriched for differentially expressed genes in the blood and brain combined are plotted according to the normalized enrichment score. Enrichment score indicates whether the genes in the pathway are upregulated (positive) or downregulated (negative) in AD. **b** Heatmap shows strength and direction of association of levels of proteins involved in vascular damage with the expression of leading-edge genes from significant pathways where enrichment scores are in opposite directions between *APOE* ε4 carriers (+) and non-carriers (-). Genes whose expression was significantly (*P* < 4.67 × 10^−4^) associated with the level of at least one protein (indicated by an asterisk) are shown

We identified 21 pathways that were significantly enriched for upregulated or downregulated genes in the combined blood and brain expression levels in at least one *APOE* genotype group (Fig. 2a and Supplementary Table 6). Enrichment scores from significant pathways identified in the ε3/ε4 group were generally downregulated and had the opposite effect direction compared to those for the other *APOE* genotype groups (Fig. 2a, Table 4). Six pathways were significantly and uniquely enriched in the ε3/ε4 subgroup including apoptosis, estrogen response late, hypoxia, il6/jak/stat3 signaling, inflammatory response, and p53 pathway. Pathways for allograft rejection, interferon gamma response, peroxisome, and TNFA signaling via NFKB were enriched for upregulated AD genes in the ε3/ε4 group but for downregulated AD genes in the ε2/ε3 and ε3/ε3 groups. There was little overlap of the leading-edge genes for these four pathways across *APOE* genotype groups with at most one overlapping gene per pathway (Table 4). Leading-edge genes in the same pathway shared by different *APOE* genotypes included *HLA-DRA* in allograft rejection, *CD74* in interferon gamma response, and *KYNU* in TNFA signaling via NFKB. Among 92 leading-edge genes from the four pathways that had enrichment scores in opposite directions among subjects with and without ε4 (Table 4), the expression of 21 genes in FHS/ADRC brains was significantly associated (*P* < 4.67 × 10^−4^) with at least one of the three vascular injury-related proteins (Fig. 2b, Table 5). The expression of 11 of these 21 genes (52%) was significantly associated with the SAA level, and the expression of 13 genes (62%) was significantly associated with the VCAM-1 level. The expression of only two genes, *TRIP10* and *FOSL1*, which are both involved in signaling via NFKB, was significantly associated with ICAM-1 levels.

**Table 4:** Significant co-expressed gene pathways in the combined blood and brain datasets

Hallmark pathway	***APOE*** genotype	NES	Adjusted ***P***-value	Leading-edge genes
Allograft rejection	ɛ3/ɛ3	−1.89	0.02	C2, HLA-DQA1, FAS, HLA-A, UBE2N, HLA-DOB, LTB, F2R, HLA-DRA, TAP2, B2M, CD1D, CD74, MAP3K7
	ɛ3/ɛ4	2.16	7.4 × 10^−3^	IRF4, CCL22, IRF7, CD74, HLA-DRA, ELF4, IL16, IFNGR2, IL27RA, IL1B
Interferon gamma response	ɛ3/ɛ3	−2.56	1.3 × 10^−5^	HLA-DQA1, FAS, CFB, BPGM, C1S, HLA-A, LAP3, MVP, PSME2, PSMA2, UBE2L6, SERPING1, DHX58, IFITM2, CD38, B2M, CD74
	ɛ3/ɛ4	1.80	0.04	CD274, CD69, BTG1, ISG20, PML, IRF4, NFKBIA, IRF7, CD74, IL10RA, IRF9
Peroxisome	ɛ3/ɛ3	−1.81	0.03	IDH2, EHHADH, MVP, ALDH1A1, SCP2, SOD1, ABCD2, MSH2
	ɛ3/ɛ4	1.90	0.03	RDH11, ELOVL5, SLC25A19, CTPS1, SLC23A2, SEMA3C
Tnfa signaling via nfkb	ɛ2/ɛ3	−2.03	0.01	DUSP4, NR4A1, NR4A3, MARCKS, NFAT5, PHLDA1, DUSP2, KYNU, G0S2, ETS2, PTGS2, GCH1, MSC, SOD2, EGR2
	ɛ3/ɛ4	2.27	2.5 × 10^−3^	TRIP10, CD69, BTG1, DENND5A, PFKFB3, FOS, NFKBIA, LDLR, IER2, JUN, IL1A, PANX1, PNRC1, DUSP1, IFNGR2, OLR1, MAFF, IL1B, TNIP2, CCL20, BIRC2, IER3, GADD45B, KYNU, LITAF, CCRL2, SPHK1, FOSL1

*NES* normalized enrichment score. Only pathways with adjusted *P*-value < 0.05 were included

**Table 5:** Association of expression of leading-edge genes from co-expressed gene networks with vascular damage protein levels

Gene	***APOE*** genotype	Pathway(s)	I-CAM1		SAA		V-CAM1	
			***β***	***P***-value	***β***	***P***-value	***β***	***P***-value
C1S	ɛ3/ɛ3	IGR	0.11	0.13	0.24	2.5 × 10^−3^	0.30	**2.0 × 10**^**−4**^
C2	ɛ3/ɛ3	AR	0.05	0.53	0.17	0.04	0.30	**2.2 × 10**^**−4**^
CD1D	ɛ3/ɛ3	AR	0.28	3.4 × 10^−3^	0.21	0.05	0.46	**1.7 × 10**^**−5**^
CD38	ɛ3/ɛ4	IGR	0.09	0.25	0.07	0.41	0.39	**6.1 × 10**^**−6**^
CFB	ɛ3/ɛ3	IGR	0.16	0.05	0.46	**2.2 × 10**^**−7**^	0.26	5.4 × 10^−3^
DHX58	ɛ3/ɛ3	IGR	0.11	0.34	0.20	0.13	0.51	**7.1 × 10**^**−5**^
ELF4	ɛ3/ɛ4	AR	0.32	8.7 × 10^−4^	0.46	**1.9 × 10**^**−5**^	0.26	0.02
FOSL1	ɛ3/ɛ4	TSN	0.20	**3.1 × 10**^**−4**^	0.18	3.1 × 10^−3^	0.08	0.21
HLA-A	ɛ3/ɛ3	AR; IGR	0.27	0.03	0.38	3.9 × 10^−3^	0.53	**7.0 × 10**^**−5**^
IDH2	ɛ3/ɛ3	P	0.09	0.43	0.24	0.05	0.61	**3.6 × 10**^**−7**^
IER3	ɛ3/ɛ4	TSN	0.14	0.06	0.37	**3.5 × 10**^**−6**^	0.07	0.43
IFITM2	ɛ3/ɛ3	IGR	0.19	0.01	0.48	**2.9 × 10**^**−9**^	0.18	0.03
IRF7	ɛ3/ɛ4	AR; IGR	0.24	9.5 × 10^−4^	0.41	**2.8 × 10**^**−7**^	0.20	0.02
ISG20	ɛ3/ɛ4	IGR	0.21	3.6 × 10^−3^	0.34	**1.9 × 10**^**−5**^	0.29	**4.4 × 10**^**−4**^
LITAF	ɛ3/ɛ4	TSN	0.27	4.1 × 10^−3^	0.39	**2.1 × 10**^**−4**^	0.43	**3.9 × 10**^**−5**^
MVP	ɛ3/ɛ3	IGR; P	0.21	0.01	0.42	**7.0 × 10**^**−6**^	0.45	**2.4 × 10**^**−6**^
PFKFB3	ɛ3/ɛ4	TSN	0.30	5.6 × 10^−4^	0.30	2.6 × 10^−3^	0.35	**4.0 × 10**^**−4**^
PML	ɛ3/ɛ4	IGR	0.28	0.02	0.47	**3.8 × 10**^**−**^	0.50	**2.0 × 10**^**−4**^
SERPING1	ɛ3/ɛ3	IGR	0.17	0.13	0.35	4.4 × 10^−3^	0.50	**4.7 × 10**^**−5**^
TNIP2	ɛ3/ɛ4	TSN	0.36	1.9 × 10^−3^	0.59	**4.7 × 10**^**−6**^	0.19	0.15
TRIP10	ɛ3/ɛ4	TSN	0.32	**2.1 × 10**^**−4**^	0.35	**3.4 × 10**^**−4**^	0.06	0.58

*I-CAM* intercellular adhesion molecule 1, *SAA* serum amyloid A, *V-CAM1* vascular cell adhesion molecule 1, *AR* allograft rejection, *IGR* interferon gamma response, *P* peroxisome, *TSN* Tnfa signaling via nfkb

Results in bold surpass the multiple testing threshold (*P* < 4.67 × 10^−4^)

### Co-expression networks common to the brain and blood

**Fig. 3:**
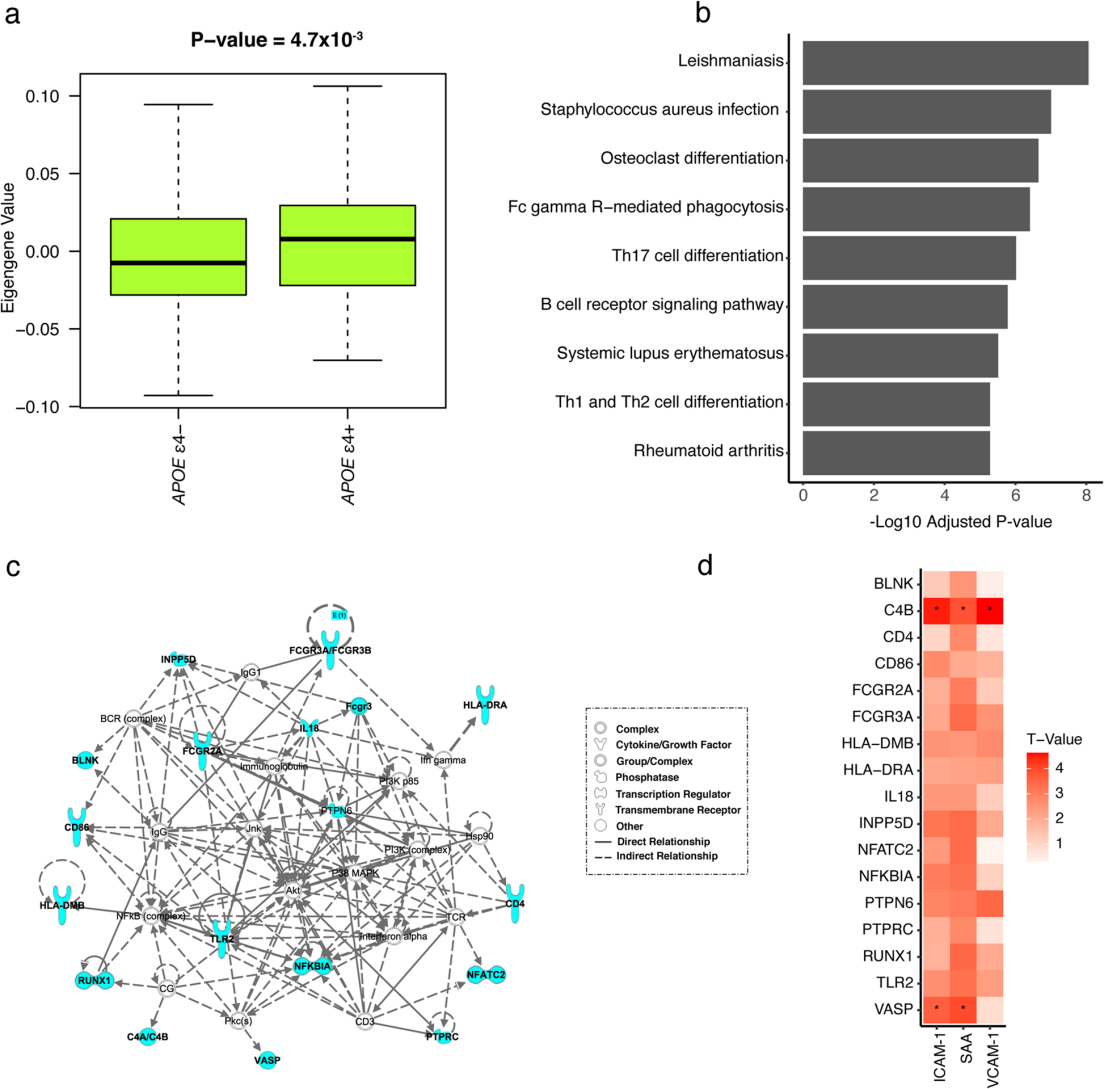
*APOE* genotype-specific co-expression networks in the blood and brain. **a** Boxplot for the light green network showing the distribution of eigengene values, which summarize gene expression across a network, among *APOE* ε4 carriers (+) and non-carriers (-). *P*-value was calculated using the Student *t*-test. **b** Barplot showing significant pathways enriched for established AD genes in the light green network. **c** Biological subnetwork including established AD genes involved in significant pathways in the light green network. **d** Heatmap showing the strength of association of seed-gene expression in the brain from the biological subnetwork in **c** with levels of proteins involved in vascular damage. Asterisks indicate significant associations (*P* < 4.67 × 10^−4^)

Four co-expression networks identified in the brain were preserved in the blood (Supplementary Table 7). The eigengene value (i.e., first principle component of gene expression across the network) in the light green network was significantly higher among ε4 carriers than non-carriers (*P* = 4.7 × 10^−3^) (Fig. 3a). The light green network is significantly enriched for genes previously associated with AD risk [38] and plaque score [39] (Supplementary Table 7). The AD-related genes in this network were significantly enriched in nine KEGG pathways and four hallmark pathways (Fig. 3b, Supplementary Tables 8, 9). Seventeen genes contributing to the significant KEGG pathways form a biological subnetwork (Fig. 3c). One of these genes, *NFKBIA*, is a leading-edge gene from the *signaling via NFKB pathway* and was involved in five out of nine significant KEGG pathways and two out of four hallmark pathways in the light green network (Table 4, Supplementary Table 8). *HLA-DRA* is involved in six of the nine significant KEGG pathways and the allograft rejection hallmark pathway in the light green network and is a leading-edge gene in the *allograft rejection pathway* identified in the ε3/ε3 and ε3/ε4 groups. *INPP5D*, which is differentially expressed in both blood and brain (Table 2, Supplementary Fig. 2), is involved in two significant KEGG pathways (*Fc gamma R-mediated phagocytosis* and *B cell receptor signaling*) in the light green network. *C4B*, which is upregulated in the brain from AD cases compared to controls in the ε2/ε3 group (Supplementary Table 5), was included in the light green network pathways involved in *Staphylococcus aureus infection* and *systemic lupus erythematosus*.

Two of the 17 subnetwork genes in the significant pathways enriched for AD genes in the light green network (Supplementary Table 10) were significantly associated with the level of at least one of the three vascular injury proteins after multiple testing correction (Fig. 3d). Specifically, *VASP* expression was significantly associated with levels of ICAM-1 (*P* = 3.7 × 10^−4^, *P*_adj_ = 0.04) and SAA (*P* = 1.0 × 10^−4^, *P*_adj_ = 0.01) and *C4B* expression was significantly associated with levels of ICAM-1 (*P* = 1.3 × 10^−5^, *P*_adj_ = 1.4 × 10^−3^), SAA (*P* = 1.6 × 10^−4^, *P*_adj_ = 0.02), and VCAM-1 (*P* = 7.0 × 10^−6^_,_ *P*_adj_ = 7.6 × 10^−4^).

## Discussion

The primary purpose of this study was to identify genes previously associated with AD and in biological pathways enriched for AD genes whose expression differs between AD cases and controls in both blood and brain, especially in an *APOE* genotype-specific manner. We observed that two established AD genes, *INPP5D* and *HLA-DQA1*, were differentially expressed in both blood and brain. Among the 21 top-ranked pathways in the combined blood and brain expression profiles, 10 pathways were specific to persons having the *APOE* ɛ3/ɛ4 genotype. Additionally, we identified a co-expression network enriched for AD genes in the brain that was preserved in the blood and showed significantly higher average expression in ε4 carriers than non-carriers. Lastly, several genes from the top-ranked pathways and co-expression networks were significantly associated with levels of vascular injury proteins. These findings suggest that AD genes that are differentially expressed in both blood and brain and associated with vascular markers, and their effects are dependent on *APOE* genotypes.

The BBB is a semi-permeable endothelial cell membrane regulating transport between cerebral blood vessels and the central nervous system [42]. The dysregulation of the BBB has been implicated in early cognitive decline and exacerbation of neuroinflammation and neurodegeneration [43]. A recent study showed that *APOE* ε4 carriers exhibit BBB dysfunction and cognitive decline independent of AD pathology [10]. Our analyses identified six pathways uniquely enriched for DEGs among ε3/ε4 carriers in combined blood and brain expression data. Expression of *INPP5D* and *HLA-DQA-1* was significantly greater in both blood and brain from individuals with AD compared to controls. Increased expression of *INPP5D* in blood has been previously linked with an increased risk of hemorrhagic transformation [44], which is associated also with BBB permeability [45]. *INPP5D* is highly expressed in microglia and encodes the protein SHIP1 which has been implicated in many neuroinflammatory processes [46]. Additionally, *HLA-DQA1* and *INPP5D* are expressed in dendritic cells and monocytes and involved in immune processes, and the migration of monocytes across an inflamed BBB can cause differentiation into dendritic cells [47]. *FRAS1* was significantly downregulated in AD compared to controls in blood from ε3/ε4 AD individuals and a recent study showed that *FRAS1* knockdown mice were impaired in memory and learning behaviors [48].

We identified four pathways (allograft rejection, interferon gamma response, peroxisome, and TNFA signaling via NFKB) containing gene sets that, with respect to AD, were significantly upregulated in the blood and brain from ε4 carriers and other gene sets from the same pathways that were downregulated in individuals without ε4. The inflammatory cytokine interferon gamma has been shown to impact directly brain endothelium to cause BBB breakdown [49] and can inhibit ApoE production in macrophages [50]. Peroxisomes synthesize fatty acids which have been implicated in the development of AD [51]. The TNFA via NFKB signaling pathway has been implicated in BBB dysfunction [52], and the TNFA and NFKB pathways have been independently associated with increased neuroinflammation related to *APOE* ε4 [53, 54].

Multiple genes from networks we observed to be preserved in the brain and blood transcriptome data and enriched in pathways from combined blood and brain expression profiles showed significant association with the vascular injury proteins ICAM-1, SAA, and VCAM-1. The SAA level increases in the presence of BBB dysfunction [55]. ICAM-1 is a cytokine involved in the regulation of the BBB [56], and increased ICAM-1 level has been associated with BBB damage and neuroinflammation [57]. Under inflammatory conditions, the VCAM-1 level is upregulated and the BBB can release soluble VCAM-1 which in turn can compromise BBB function [58]. Our study showed that *FOSL1* and *TRIP10* were among the genes enriched in the TNFA via the NFKB pathway, and their expression was associated with ICAM-1. *TRIP10* was previously included in an AD network derived from multi-omic integration [59] and *FOSL-1* was identified in conjunction with PIAS1, a protein associated with AD and inflammatory response [60]. We identified *VASP* and *C4B* in an *APOE* genotype-specific co-expressed gene network in the brain that was reproduced in the blood, and the expression of these genes was significantly associated with levels of multiple vascular damage proteins. *VASP* encodes vasodilator-stimulated phosphoprotein which regulates BBB function [61]. Additionally, *VASP* has been recently implicated in a microglial network in AD [62]. The pattern of *C4B* expression in the brain is dependent on the *APOE* genotype [19], and dysregulation of the complement system can cause or exacerbate BBB dysfunction [63]. C4B-binding-protein levels in cerebral spinal fluid have been shown to correlate with BBB integrity [64]. These genes require further investigation in their role with AD specifically related to BBB function and *APOE*.

### Limitations

Our study has several limitations. First, the sample sizes of the *APOE* genotype groups in the blood dataset were relatively small which limited statistical power. Additionally, the ROSMAP blood dataset exhibited significant batch effects. However, we were able to account for these batch effects by running each batch separately and meta-analyzing our results. Second, the software WGCNA creates networks based on strong computational correlations but does not account for underlying biological implications. We evaluated biological connections using the IPA software by rebuilding subnetworks of the leading-edge genes. Third, publicly available single-cell data were available only for dendritic cells and monocytes in the blood, and therefore, we could not analyze a wider array of blood cell types. Fourth, we were unable to account for RIN in the ROSMAP blood dataset because this information was unavailable. Fifth, because expression profiles may differ between tissues, lack of overlap between brain and blood does not necessarily exclude the relevance of some of our discordant findings across tissues to AD and BBB dysfunction. Finally, although there was little overlap of leading-edge genes in GSEA pathways across *APOE* genotype groups, several particular pathways containing a different complement of genes were significant among individuals with different *APOE* genotypes. Experimental studies are needed to confirm the mechanisms involving these genes.

## Conclusions

Our study provides evidence of the importance of evaluating brain and blood transcriptome data together with genetic information derived from the same subjects to identify meaningful correlations of blood biomarkers with AD-related proteins in the brain. Future studies are required to investigate further, how the genes and biological pathways identified in this study in the context of the *APOE* genotype influence the BBB and contribute to and/or exacerbate AD-related pathology.

## Supplementary Information

**Additional file 1.** Supplementary Figs. 1–4 and Supplementary Tables 1–10.

## Data Availability

The datasets supporting the conclusions of this article are available in https://singlecell.broadinstitute.org/ and http://www.synapse.org, as well as from the corresponding author upon request.

## Abbreviations

Aβ: Amyloid-β
AD: Alzheimer’s disease
BBB: Blood-brain barrier
CAA: Cerebral amyloid angiopathy
CERAD: Consortium to Establish a Registry for Alzheimer Disease
CPM: Counts per million
DE: Differential expression
DGE: Differential gene expression
FHS/ADRC: Framingham Heart Study/Boston University Alzheimer’s Disease Research Center
FPKM: Fragments per kilobase of transcript per million
GWAS: Genome-wide association study
ICAM-1: Intercellular adhesion molecule 1
PMI: Post-mortem interval
RIN: RNA integrity number
ROSMAP: Religious Orders Study and Rush Memory and Aging Project
RNA-seq: RNA sequencing
SAA: Serum amyloid α
VCAM-1: Vascular cell adhesion molecule 1
WGCNA: Weighted gene correlation network analysis
