# Supplementary material for: Blood and brain transcriptome analysis reveals APOE genotype-mediated and immune-related pathways involved in Alzheimer disease
Source: Alzheimers Res Ther. 2022 Feb 9;14:30. doi: 10.1186/s13195-022-00975-z (PMC8830081; doi:10.1186/s13195-022-00975-z)
Supplement: Supplementary file 1 — Additional file 1. Supplementary Figs. 1–4 and Supplementary Tables 1–10. [file 13195_2022_975_MOESM1_ESM.docx]

**SUPPLEMENTARY INFORMATION**

**Supplementary Figure 1.** Uniform Manifold Approximation and Projection of (a) Blood RNA-seq Samples and (b) Brain RNA-seq Samples by batch. RNA batch was determined in the initial RNA processing steps and show separation in the data.

**a.**

**b.**

**Supplementary Figure 2.** Expression of AD-related Genes in Blood and Brain. Heatmaps show differential expression of genes from Tables 1 and 2 between AD cases and controls by *APOE* genotype in blood, brain, and the combined datasets. NA: not available due to low expression filtering. Asterisks indicate P<0.05.


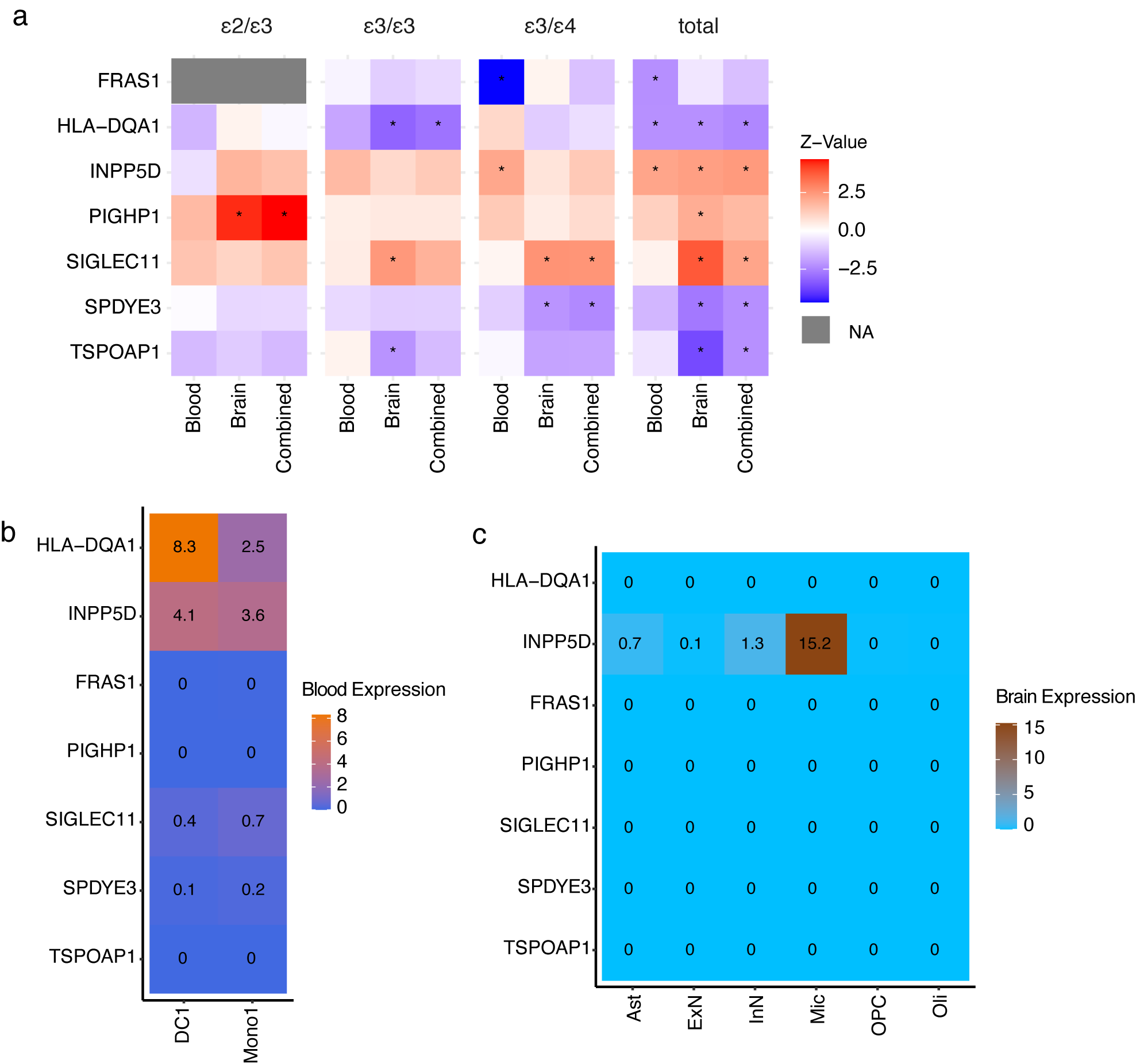


**Supplementary Figure 3.** Boxplots of normalized expression with differential expression p-values between AD and control individuals of (a) *PIGHP1* and (b) *FRAS1* by *APOE* genotype in blood and brain in the ROSMAP dataset. NA: not available due to low expression.


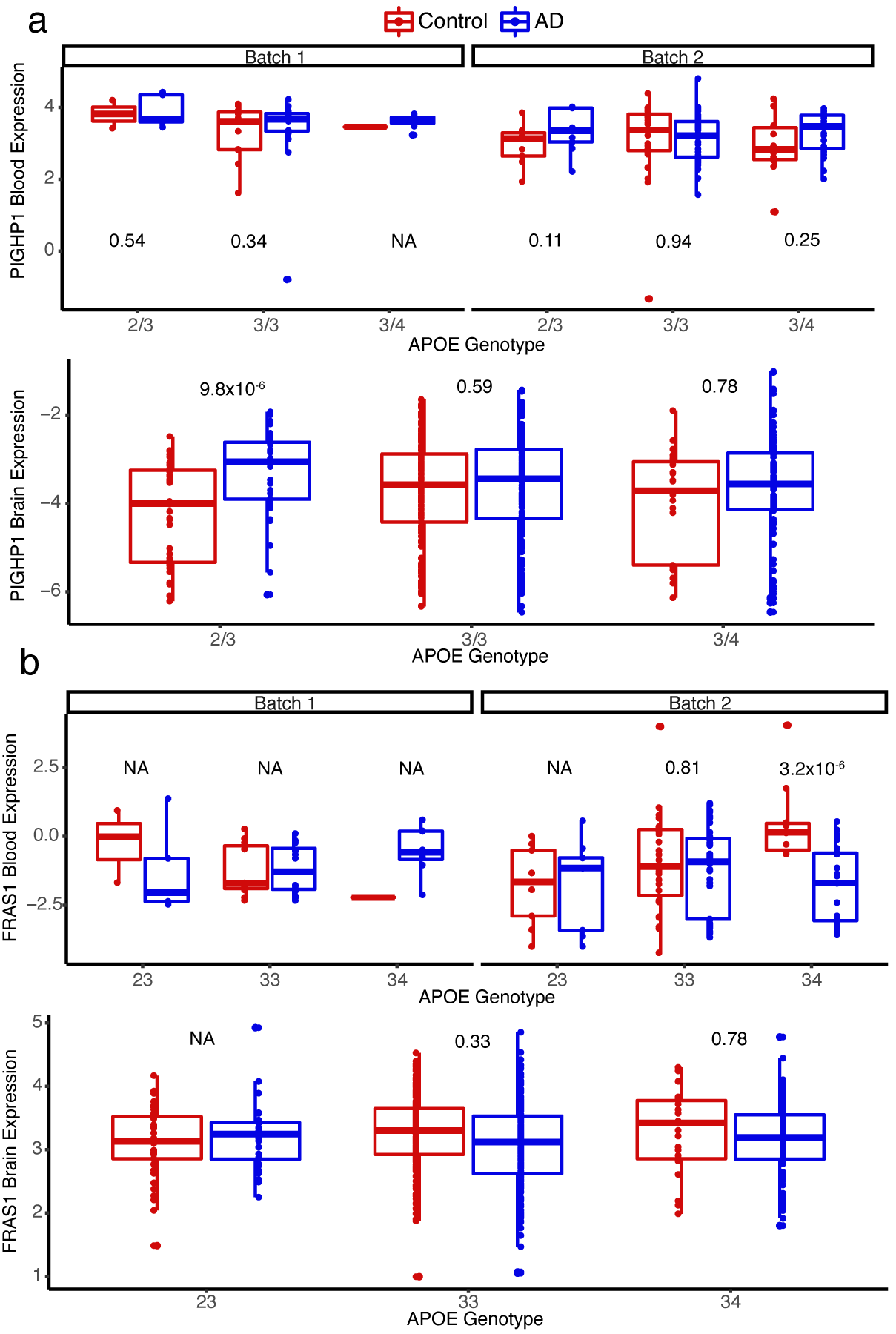


**Supplementary Figure 4.** Single-cell expression of AD-associated genes (from Tables 1 and 2) in blood and brain. (**a)** Heatmap depicting the average single cell expression in dendritic cells (DC) and monocytes (Mono) derived from blood. **(b)** Heatmap depicting average single nuclei expression in brain related cells including astrocytes (ast), excitatory neurons (ExN), inhibitory neurons (InN), microglia (Mic), oligodendrocyte progenitor cells (OPC), and oligodendrocytes (Oli).

b

a


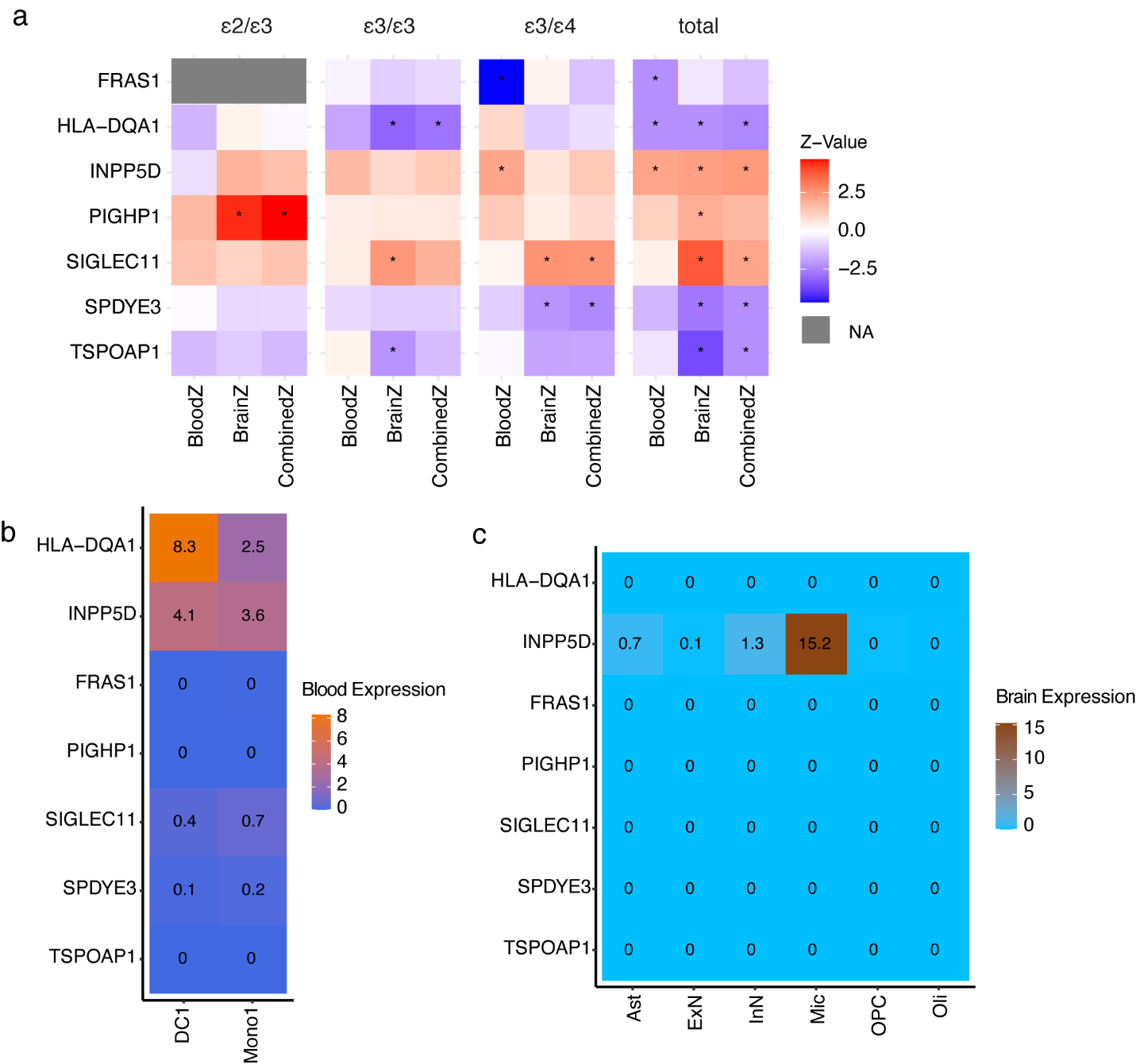


**Supplementary Table 1.** Number of ROSMAP subjects with RNA-seq data by *APOE* genotype and batch

| **APOE**  **Genotype** |  | **Blood Batch 1** |  | **Blood Batch 2** |  | **Blood Batch 3*** |  | **Brain** |
| --- | --- | --- | --- | --- | --- | --- | --- | --- |
| ɛ2/ɛ2 |  | 0 |  | 1 |  | 0 |  | 5 |
| ɛ2/ɛ3 |  | 8 |  | 18 |  | 0 |  | 82 |
| ɛ3/ɛ3 |  | 27 |  | 75 |  | 0 |  | 386 |
| ɛ2/ɛ4 |  | 2 |  | 5 |  | 0 |  | 16 |
| ɛ3/ɛ4 |  | 9 |  | 31 |  | 0 |  | 142 |
| ɛ4/ɛ4 |  | 1 |  | 1 |  | 0 |  | 6 |
| NA |  | 0 |  | 70 |  | 366 |  | 2 |
| **TOTAL** |  | 47 |  | 201 |  | 366 |  | 639 |

Blood Batch 3 was excluded from analysis because it did not contain any neuropathological data.

**Supplementary Table 2.** Differential expression analysis sample sizes in brain by *APOE* genotype and batch

| **Brain Batch** |  | **ɛ2/ɛ3** | | |  | **ɛ3/ɛ3** | | |  | **ɛ3/ɛ4** | | |
| --- | --- | --- | --- | --- | --- | --- | --- | --- | --- | --- | --- | --- |
|  |  | **AD** |  | **CTRL** |  | **AD** |  | **CTRL** |  | **AD** |  | **CTRL** |
| 0 |  | 0 |  | 0 |  | 2 |  | 7 |  | 1 |  | 1 |
| 1 |  | 8 |  | 13 |  | 19 |  | 31 |  | 8 |  | 6 |
| 2 |  | 3 |  | 4 |  | 35 |  | 27 |  | 15 |  | 3 |
| 3 |  | 5 |  | 5 |  | 29 |  | 18 |  | 19 |  | 3 |
| 4 |  | 2 |  | 6 |  | 30 |  | 15 |  | 16 |  | 3 |
| 5 |  | 4 |  | 6 |  | 27 |  | 23 |  | 16 |  | 5 |
| 6 |  | 4 |  | 4 |  | 32 |  | 26 |  | 18 |  | 0 |
| 7 |  | 3 |  | 1 |  | 13 |  | 5 |  | 7 |  | 2 |
| 8 |  | 3 |  | 0 |  | 10 |  | 6 |  | 1 |  | 2 |

AD: AD cases; CTRL: controls

**Supplementary Table 3.** Co-Expression Network Analysis Sample Sizes

| **APOE**  **Genotype** |  | **Blood** | | |  | **Brain** | | |
| --- | --- | --- | --- | --- | --- | --- | --- | --- |
|  |  | **AD** |  | **CTRL** |  | **AD** |  | **CTRL** |
| ɛ2/ɛ2 |  | 0 |  | 1 |  | 0 |  | 5 |
| ɛ2/ɛ3 |  | 9 |  | 9 |  | 38 |  | 46 |
| ɛ3/ɛ3 |  | 48 |  | 35 |  | 216 |  | 168 |
| ɛ2/ɛ4 |  | 5 |  | 0 |  | 12 |  | 4 |
| ɛ3/ɛ4 |  | 22 |  | 11 |  | 113 |  | 27 |
| ɛ4/ɛ4 |  | 1 |  | 0 |  | 5 |  | 1 |
| TOTAL |  | 85 |  | 56 |  | 385 |  | 251 |

Blood dataset includes all samples from Blood Batch 2 including samples without post-mortem AD diagnosis. AD diagnosis in blood is based on clinical evaluation. Brain dataset includes all samples including those excluded previously due to lack of RNA integrity number (RIN).

| **Supplementary Table 4.** Differential expression of AD-associated genes in blood and brain | | | | | | | | | | | |
| --- | --- | --- | --- | --- | --- | --- | --- | --- | --- | --- | --- |
| **Gene** | **Blood** | | |  | **Brain** | | |  | **Combined** | | |
|  | **N** | **Z-score** | **P-value** |  | **N** | **Z-score** | **P-value** |  | **N** | **Z-score** | **P-value** |
| *HLA-DQA1* | 179 | -2.24 | 2.54E-02 |  | 576 | -2.24 | 2.54E-02 |  | 755 | -2.42 | 1.54E-02 |
| *INPP5D* | 179 | 2.16 | 3.09E-02 |  | 576 | 2.30 | 2.13E-02 |  | 755 | 2.42 | 1.57E-02 |
| *SPDYE3* | 179 | -1.47 | 1.43E-01 |  | 576 | -2.73 | 6.36E-03 |  | 755 | -2.26 | 2.41E-02 |
| *TSPOAP1* | 179 | -0.55 | 5.82E-01 |  | 576 | -3.65 | 2.58E-04 |  | 755 | -2.24 | 2.54E-02 |
| *SIGLEC11* | 179 | 0.33 | 7.44E-01 |  | 576 | 3.77 | 1.64E-04 |  | 755 | 2.17 | 2.98E-02 |
| *WDR81* | 179 | -0.39 | 6.98E-01 |  | 576 | 3.26 | 1.10E-03 |  | 755 | 1.51 | 1.32E-01 |
| *EED* | 179 | 0.77 | 4.44E-01 |  | 576 | 1.93 | 5.30E-02 |  | 755 | 1.45 | 1.48E-01 |
| *ABCA7* | 179 | 0.80 | 4.23E-01 |  | 576 | 1.87 | 6.17E-02 |  | 755 | 1.43 | 1.52E-01 |
| *MYO15A* | 179 | -0.88 | 3.77E-01 |  | 576 | -1.67 | 9.48E-02 |  | 755 | -1.37 | 1.70E-01 |
| *ADAM17* | 179 | -0.49 | 6.23E-01 |  | 576 | -1.87 | 6.14E-02 |  | 755 | -1.26 | 2.07E-01 |
| *SEC61G* | 179 | -0.24 | 8.10E-01 |  | 576 | -2.06 | 3.90E-02 |  | 755 | -1.22 | 2.21E-01 |
| *MINDY2* | 179 | -0.18 | 8.58E-01 |  | 576 | 2.51 | 1.22E-02 |  | 755 | 1.22 | 2.21E-01 |
| *TNIP1* | 179 | -1.26 | 2.07E-01 |  | 576 | 3.41 | 6.58E-04 |  | 755 | 1.10 | 2.72E-01 |
| *RBCK1* | 179 | 0.23 | 8.16E-01 |  | 576 | 1.64 | 1.00E-01 |  | 755 | 1.00 | 3.18E-01 |
| *APP* | 179 | 0.04 | 9.72E-01 |  | 576 | -1.93 | 5.42E-02 |  | 755 | -1.00 | 3.19E-01 |
| *SCIMP* | 179 | 0.24 | 8.10E-01 |  | 576 | 1.61 | 1.08E-01 |  | 755 | 0.98 | 3.26E-01 |
| *SORT1* | 179 | 0.31 | 7.58E-01 |  | 576 | 1.49 | 1.35E-01 |  | 755 | 0.96 | 3.37E-01 |
| *NCK2* | 179 | -0.46 | 6.48E-01 |  | 576 | 2.28 | 2.25E-02 |  | 755 | 0.95 | 3.42E-01 |
| *ICA1* | 179 | 0.99 | 3.24E-01 |  | 576 | -2.83 | 4.69E-03 |  | 755 | -0.94 | 3.45E-01 |
| *CR1* | 179 | 0.53 | 5.99E-01 |  | 576 | 1.22 | 2.21E-01 |  | 755 | 0.94 | 3.48E-01 |
| *USP6NL* | 179 | -0.82 | 4.13E-01 |  | 576 | -0.90 | 3.71E-01 |  | 755 | -0.93 | 3.54E-01 |
| *LILRB2* | 179 | -0.98 | 3.28E-01 |  | 576 | -0.67 | 5.01E-01 |  | 755 | -0.90 | 3.69E-01 |
| *UMAD1* | 179 | 0.55 | 5.80E-01 |  | 576 | -2.22 | 2.62E-02 |  | 755 | -0.87 | 3.86E-01 |
| *BCKDK* | 179 | 1.94 | 5.21E-02 |  | 576 | -3.48 | 5.08E-04 |  | 755 | -0.76 | 4.49E-01 |
| *GRN* | 179 | -0.38 | 7.01E-01 |  | 576 | 1.83 | 6.67E-02 |  | 755 | 0.76 | 4.50E-01 |
| *CTSB* | 179 | 0.00 | 9.99E-01 |  | 576 | -1.43 | 1.54E-01 |  | 755 | -0.75 | 4.51E-01 |
| *MAF* | 179 | -0.29 | 7.72E-01 |  | 576 | 1.69 | 9.05E-02 |  | 755 | 0.73 | 4.64E-01 |
| *IDUA* | 179 | -0.98 | 3.25E-01 |  | 576 | -0.32 | 7.51E-01 |  | 755 | -0.71 | 4.75E-01 |
| *PRKD3* | 179 | -0.40 | 6.86E-01 |  | 576 | 1.75 | 7.95E-02 |  | 755 | 0.70 | 4.83E-01 |
| *TMEM106B* | 179 | 0.21 | 8.38E-01 |  | 576 | -1.50 | 1.34E-01 |  | 755 | -0.68 | 4.98E-01 |
| *RHOH* | 179 | -0.46 | 6.45E-01 |  | 576 | -0.78 | 4.37E-01 |  | 755 | -0.67 | 5.05E-01 |
| *CLU* | 179 | 0.11 | 9.16E-01 |  | 576 | 1.01 | 3.15E-01 |  | 755 | 0.59 | 5.55E-01 |
| *TPCN1* | 179 | 1.45 | 1.47E-01 |  | 576 | -0.41 | 6.79E-01 |  | 755 | 0.59 | 5.56E-01 |
| *SHARPIN* | 179 | 0.11 | 9.10E-01 |  | 576 | 0.99 | 3.20E-01 |  | 755 | 0.59 | 5.57E-01 |
| *ANKH* | 179 | 1.52 | 1.28E-01 |  | 576 | -0.50 | 6.19E-01 |  | 755 | 0.58 | 5.60E-01 |
| *SNX1* | 179 | -0.32 | 7.47E-01 |  | 576 | 1.43 | 1.51E-01 |  | 755 | 0.58 | 5.63E-01 |
| *WDR12* | 179 | 0.77 | 4.43E-01 |  | 576 | -1.90 | 5.76E-02 |  | 755 | -0.58 | 5.64E-01 |
| *CASS4* | 179 | 0.93 | 3.55E-01 |  | 576 | 0.11 | 9.09E-01 |  | 755 | 0.57 | 5.66E-01 |
| *KLF16* | 179 | 0.05 | 9.64E-01 |  | 576 | 1.04 | 2.99E-01 |  | 755 | 0.57 | 5.66E-01 |
| *ABCA1* | 179 | -0.05 | 9.63E-01 |  | 576 | 1.10 | 2.71E-01 |  | 755 | 0.56 | 5.79E-01 |
| *PTK2B* | 179 | 0.96 | 3.39E-01 |  | 576 | -2.05 | 4.05E-02 |  | 755 | -0.55 | 5.82E-01 |
| *EPHA1* | 179 | -0.33 | 7.41E-01 |  | 576 | 1.34 | 1.82E-01 |  | 755 | 0.52 | 6.02E-01 |
| *MS4A4A* | 179 | 1.52 | 1.28E-01 |  | 576 | -0.68 | 4.97E-01 |  | 755 | 0.49 | 6.26E-01 |
| *BLNK* | 132 | 1.25 | 2.11E-01 |  | 576 | -0.17 | 8.68E-01 |  | 708 | 0.46 | 6.46E-01 |
| *BIN1* | 179 | -0.63 | 5.26E-01 |  | 576 | 1.51 | 1.30E-01 |  | 755 | 0.45 | 6.55E-01 |
| *FERMT2* | 132 | 0.41 | 6.81E-01 |  | 576 | -1.09 | 2.74E-01 |  | 708 | -0.43 | 6.67E-01 |
| *SPPL2A* | 179 | -0.78 | 4.38E-01 |  | 576 | 0.05 | 9.61E-01 |  | 755 | -0.41 | 6.85E-01 |
| *JAZF1* | 179 | 1.15 | 2.51E-01 |  | 576 | -1.97 | 4.91E-02 |  | 755 | -0.40 | 6.88E-01 |
| *ABI3* | 179 | -0.14 | 8.89E-01 |  | 576 | 0.77 | 4.38E-01 |  | 755 | 0.33 | 7.40E-01 |
| *SORL1* | 179 | 1.32 | 1.88E-01 |  | 576 | -1.98 | 4.81E-02 |  | 755 | -0.31 | 7.55E-01 |
| *SORL1* | 179 | 1.32 | 1.88E-01 |  | 576 | -1.98 | 4.81E-02 |  | 755 | -0.31 | 7.55E-01 |
| *TSPAN14* | 179 | -1.42 | 1.57E-01 |  | 576 | 2.06 | 3.95E-02 |  | 755 | 0.30 | 7.64E-01 |
| *SLC2A4RG* | 179 | 0.45 | 6.54E-01 |  | 576 | 0.09 | 9.32E-01 |  | 755 | 0.29 | 7.69E-01 |
| *MME* | 132 | -0.54 | 5.90E-01 |  | 576 | -0.07 | 9.42E-01 |  | 708 | -0.28 | 7.81E-01 |
| *APH1B* | 179 | 0.40 | 6.86E-01 |  | 576 | 0.09 | 9.26E-01 |  | 755 | 0.27 | 7.85E-01 |
| *COX7C* | 179 | 0.09 | 9.25E-01 |  | 576 | -0.53 | 5.95E-01 |  | 755 | -0.23 | 8.19E-01 |
| *PLCG2* | 179 | 0.58 | 5.63E-01 |  | 576 | -0.26 | 7.95E-01 |  | 755 | 0.18 | 8.54E-01 |
| *CTSH* | 179 | 0.35 | 7.26E-01 |  | 576 | -0.05 | 9.64E-01 |  | 755 | 0.17 | 8.65E-01 |
| *CD2AP* | 179 | -0.36 | 7.19E-01 |  | 576 | 0.65 | 5.13E-01 |  | 755 | 0.15 | 8.84E-01 |
| *SPI1* | 179 | -1.22 | 2.24E-01 |  | 576 | 1.01 | 3.11E-01 |  | 755 | -0.14 | 8.88E-01 |
| *SLC24A4* | 179 | 0.72 | 4.70E-01 |  | 576 | -0.55 | 5.85E-01 |  | 755 | 0.11 | 9.10E-01 |
| *SLC24A4* | 179 | 0.72 | 4.70E-01 |  | 576 | -0.55 | 5.85E-01 |  | 755 | 0.11 | 9.10E-01 |
| *ANK3* | 132 | 0.60 | 5.51E-01 |  | 576 | -0.64 | 5.20E-01 |  | 708 | -0.10 | 9.24E-01 |
| *PLEKHA1* | 179 | 0.29 | 7.71E-01 |  | 576 | -0.21 | 8.31E-01 |  | 755 | 0.05 | 9.61E-01 |
| *UNC5CL* | 179 | -0.77 | 4.42E-01 |  | 576 | 0.90 | 3.70E-01 |  | 755 | 0.05 | 9.63E-01 |

**Supplementary Table 5.** Differential expression of previously reported *APOE* ɛ2/ɛ3 network AD-related genes in the ROSMAP dataset

| **Gene** | **Dataset** | ***APOE* ɛ2/ɛ3** | | |  | ***APOE*** **ɛ3/ɛ3** | | |  | ***APOE*** **ɛ3/ɛ4** | | |  | **Total** | | | |
| --- | --- | --- | --- | --- | --- | --- | --- | --- | --- | --- | --- | --- | --- | --- | --- | --- | --- |
|  |  | **N** | **Z** | **P** |  | **N** | **Z** | **P** |  | **N** | **Z** | **P** |  | **N** | **Z** | **P** |  |
| *C4B* | Blood | 18 | -1.77 | 0.08 |  | 75 | -0.77 | 0.44 |  | 31 | 0.47 | 0.64 |  | 132 | -1.13 | 0.26 |  |
|  | Brain | 71 | 3.45 | 5.6x10^-4^ |  | 355 | 0.69 | 0.49 |  | 126 | 1.13 | 0.26 |  | 576 | 2.79 | 5.2x10^-4^ |  |
|  | Combined | 89 | 2.93 | 3.4x10^-3^ |  | 430 | 0.26 | 0.79 |  | 157 | 1.23 | 0.22 |  | 708 | 1.06 | 0.28 |  |
| *C4A* | Blood | 18 | -0.02 | 0.98 |  | 75 | 1.16 | 0.24 |  | 31 | 0.03 | 0.98 |  | 132 | 0.71 | 0.48 |  |
|  | Brain | 71 | 3.40 | 6.7x10^-4^ |  | 355 | 0.17 | 0.86 |  | 126 | 0.59 | 0.55 |  | 576 | 2.81 | 4.9x10^-3^ |  |
|  | Combined | 89 | 3.28 | 1.0x10^-3^ |  | 430 | 0.47 | 0.64 |  | 157 | 0.58 | 0.56 |  | 708 | 1.88 | 0.06 |  |
| *HSPA2* | Blood | 18 | 0.71 | 0.48 |  | 102 | -0.18 | 0.86 |  | 31 | 0.13 | 0.89 |  | 179 | -0.44 | 0.66 |  |
|  | Brain | 71 | 1.58 | 0.11 |  | 355 | 1.89 | 0.06 |  | 126 | 1.18 | 0.24 |  | 576 | 3.82 | 1.3x10^-4^ |  |
|  | Combined | 89 | 1.68 | 0.09 |  | 457 | 1.21 | 0.23 |  | 157 | 1.17 | 0.24 |  | 755 | 1.7 | 0.09 |  |

**Supplementary Table 6.** Leading-edge genes in significant pathways

| **Hallmark Pathway** |  | **Group** |  | **NES** |  | **AdjP** |  | **Leading Edge Genes** |
| --- | --- | --- | --- | --- | --- | --- | --- | --- |
| Allograft rejection |  | E33 |  | -1.89 |  | 0.02 |  | C2, HLA-DQA1, FAS, HLA-A, UBE2N, HLA-DOB, LTB, F2R, HLA-DRA, TAP2, B2M, CD1D, CD74, MAP3K7 |
|  |  | E34 |  | 2.16 |  | 7.4x10^-3^ |  | IRF4, CCL22, IRF7, CD74, HLA-DRA, ELF4, IL16, IFNGR2, IL27RA, IL1B |
| Apoptosis |  | E34 |  | 1.87 |  | 0.04 |  | SMAD7, PEA15, CD69, BCL2L11, ISG20, BMF, JUN, IL1A, BIK, IL1B, AVPR1A, IER3, GADD45B, FEZ1 |
| Complement |  | E33 |  | -2.24 |  | 1.9x10^-3^ |  | C2, S100A9, ERAP2, CFB, SERPINA1, C1S, PDP1, LAP3, APOBEC3G, CALM3, PREP, CP, SERPING1, VCPIP1, ATOX1, GPD2, CDH13, AKAP10, LGMN, ADAM9 |
| Estrogen response late |  | E34 |  | 1.93 |  | 0.03 |  | ISG20, TOB1, ELOVL5, FOS, SLC9A3R1 |
| Fatty acid metabolism |  | E33 |  | -1.78 |  | 0.03 |  | BCKDHB, HMGCS1, SMS, SDHD, EHHADH, DECR1, ALDH1A1, MAOA, UBE2L6, IDH3B, CD1D, GPD2 |
| Hypoxia |  | E34 |  | 1.77 |  | 0.04 |  | BTG1, ISG20, PFKFB3, MXI1, FOS, JUN, SLC2A5, HK2, PNRC1, DUSP1, ADM, MAFF, P4HA2, PFKP, ERRFI1, IER3 |
| Il2 stat5 signaling |  | E33 |  | -1.80 |  | 0.03 |  | CD48, BATF3, AHCY, PTRH2, CKAP4, LTB, SHE, TTC39B, PTGER2, MAP3K8, LRIG1, TRAF1, NRP1, SNX14 |
| Il6 jak stat3 signaling |  | E34 |  | 1.80 |  | 0.05 |  | JUN, BAK1, IRF9, IFNGR2, IL1B, CBL, IL3RA |
| Inflammatory response |  | E34 |  | 2.27 |  | 2.5x10^-3^ |  | LPAR1, CD69, NOD2, RAF1, NFKBIA, LDLR, CCL22, IRF7, IL1A, P2RX7, IL10RA, IFITM1, ADM, PTAFR, IFNGR2, OLR1, IL1B, CCL20, MSR1, CCRL2, SLC31A2, SPHK1, CLEC5A, PTGER2 |
| Interferon alpha response |  | E23 |  | 1.96 |  | 0.02 |  | IFITM2, CNP, MVB12A, CMTR1, PSMB8, IFI35, C1S |
|  |  | E33 |  | -2.19 |  | 4.1x10^-3^ |  | C1S, LAP3, IFITM1, PSME2, UBE2L6, DHX58, IFITM2, B2M, PROCR, CD74 |
| Interferon gamma response |  | E33 |  | -2.56 |  | 1.3x10^-5^ |  | HLA-DQA1, FAS, CFB, BPGM, C1S, HLA-A, LAP3, MVP, PSME2, PSMA2, UBE2L6, SERPING1, DHX58, IFITM2, CD38, B2M, CD74 |
|  |  | E34 |  | 1.80 |  | 0.04 |  | CD274, CD69, BTG1, ISG20, PML, IRF4, NFKBIA, IRF7, CD74, IL10RA, IRF9 |
| Mitotic spindle |  | E33 |  | 1.81 |  | 0.03 |  | RALBP1, CCDC88A, PREX1, NUMA1, ARHGAP5, DLG1, PCM1, MYO9B, HOOK3, ACTN4, CNTRL, CNTROB |
|  |  | all |  | 1.79 |  | 0.04 |  | SMC1A, MYO1E, CDC42BPA, SMC3, DLG1, CDK5RAP2, PALLD, BCL2L11, NUMA1, ACTN4, MYH9, KATNA1, ARHGAP27, INCENP, TUBD1 |
| Mtorc1 signaling |  | E33 |  | -1.87 |  | 0.01 |  | HMGCS1, RAB1A, HPRT1, MCM4, GOT1, CALR, SDF2L1, PSMC2, MLLT11, SRD5A1, GSR, EEF1E1, LDLR, PSMB5, SKAP2, MCM2, UCHL5, LGMN |
| Myc targets v1 |  | E23 |  | -2.21 |  | 4.5x10^-3^ |  | PRDX3, EIF4E, HNRNPC, SSBP1, SET, PSMC6, TOMM70, DUT, RAN, CCT4, TXNL4A, TRA2B, PSMA2, SNRPD1, EIF2S1, RANBP1, HSP90AB1, SRSF1, C1QBP, XPOT, VDAC1, GOT2, HDAC2, HSPD1, NHP2, NDUFAB1, HSPE1 |
| Myc targets v1 |  | E33 |  | -2.19 |  | 4.1x10^-3^ |  | HPRT1, CCT4, NHP2, MCM4, HNRNPR, ERH, MAD2L1, PSMA2, DUT, NDUFAB1, GOT2, TOMM70 |
| Oxidative phosphorylation |  | E23 |  | -2.20 |  | 4.5x10^-3^ |  | FDX1, SUCLA2, ATP5PB, SDHC, PRDX3, PDHB, ATP6V1H, NDUFB3, MFN2, MRPS30, ATP6V1C1, NDUFB2, TOMM70, IDH3B, LRPPRC, AFG3L2, NDUFS4, NDUFB6, NNT, MDH2, SDHB, MTRF1, COX11, IDH3A, PHYH, PDHA1, NDUFS6, UQCRH, NDUFC2, UQCR11, VDAC1, UQCRC2, HTRA2, GOT2, MDH1, ATP5MG |
|  |  | E33 |  | -2.65 |  | 2.9x10^-6^ |  | ATP5PB, NDUFA5, TIMM8B, MRPS22, ATP6V1H, SDHD, IDH2, ATP5MF, PDP1, DECR1, GLUD1, MRPS30, NDUFA6, DLAT, UQCRFS1, NDUFAB1, NDUFB4, GOT2, ETFA, TOMM70, NDUFC1, COX7B, IDH3B, ATP1B1, NDUFC2, CYC1 |
|  |  | all |  | -1.88 |  | 0.04 |  | ATP6V1H, MRPS22, DLAT, TOMM70, ATP6V1D, ATP1B1, IDH3B, SDHD, TIMM17A, ACAA2, NDUFC1, NDUFB5, ETFB, UQCRC2, SDHB, CYCS, ATP6V1G1, VDAC1, PDHX |
| P53 pathway |  | E34 |  | 2.35 |  | 2.5x10^-3^ |  | ABHD4, CDKN2AIP, SP1, CEBPA, STEAP3, BTG1, TOB1, FOS, SERTAD3, JUN, IL1A, COQ8A, BAK1, WWP1 |
| Peroxisome |  | E33 |  | -1.81 |  | 0.03 |  | IDH2, EHHADH, MVP, ALDH1A1, SCP2, SOD1, ABCD2, MSH2 |
|  |  | E34 |  | 1.90 |  | 0.03 |  | RDH11, ELOVL5, SLC25A19, CTPS1, SLC23A2, SEMA3C |
| Reactive oxygen species pathway |  | E33 |  | -2.12 |  | 9.3x10^-3^ |  | GCLM, NDUFA6, SOD1, GSR, NDUFB4, PRDX2, ATOX1, SRXN1 |
| Tnfa signaling via nfkb |  | E23 |  | -2.03 |  | 0.01 |  | DUSP4, NR4A1, NR4A3, MARCKS, NFAT5, PHLDA1, DUSP2, KYNU, G0S2, ETS2, PTGS2, GCH1, MSC, SOD2, EGR2 |
|  |  | E34 |  | 2.27 |  | 2.5x10^-3^ |  | TRIP10, CD69, BTG1, DENND5A, PFKFB3, FOS, NFKBIA, LDLR, IER2, JUN, IL1A, PANX1, PNRC1, DUSP1, IFNGR2, OLR1, MAFF, IL1B, TNIP2, CCL20, BIRC2, IER3, GADD45B, KYNU, LITAF, CCRL2, SPHK1, FOSL1 |
| Xenobiotic metabolism |  | E33 |  | -2.08 |  | 4.1x10^-3^ |  | MTHFD1, FAS, CFB, ENTPD5, PROS1, HPRT1, PGRMC1, MAOA, ALDH2, AHCY, ABCD2, GSR, ASL, SLC35B1 |

**Supplementary Table 7.** Preserved co-expressed gene networks in brain and blood that are enriched for established AD risk genes

| Network | Z-summary | Total # of Genes | Trait | | | | | | | | |
| --- | --- | --- | --- | --- | --- | --- | --- | --- | --- | --- | --- |
|  |  |  | AD Risk | | |  | Neurofibrillary Tangles | |  | Amyloid-β Plaques | |
|  |  |  | # of Genes | P-value_adj_ |  | | # of Genes | P-value_adj_ |  | # of Genes | P-value_adj_ |
| blue | 8.0 | 2,297 | 530 | 1.8x10^-5^ |  | | 182 | 0.01 |  | 158 | 0.25 |
| green | 6.1 | 1,044 | 213 | 0.43 |  | | 92 | 6.9x10^-3^ |  | 69 | 0.50 |
| light green | 5.8 | 230 | 64 | 2.8x10^-3^ |  | | 20 | 0.17 |  | 25 | 8.6x10^-3^ |
| turquoise | 5.1 | 4,169 | 751 | 1.00 |  | | 241 | 1.00 |  | 256 | 0.95 |

**Supplementary Table 8.** Significant KEGG pathways enriched for AD risk genes in the light green network

| Pathway | Overlap | P-value | Adjusted P-value | Genes |
| --- | --- | --- | --- | --- |
| Leishmaniasis | 8/74 | 8.9x10^-11^ | 8.7x10^-9^ | NFKBIA;FCGR3A;HLA-DMA;HLA-DMB;FCGR2A;HLA-DRA;PTPN6;TLR2 |
| Staphylococcus aureus infection | 7/68 | 2.0x10^-9^ | 9.9x10^-8^ | C4B;FCGR3A;HLA-DMA;HLA-DMB;FCGR2A;C5AR1;HLA-DRA |
| Osteoclast differentiation | 8/127 | 7.0x10^-9^ | 2.3x10^-7^ | NFKBIA;FCGR3A;SPI1;FCGR2A;BLNK;NFATC2;TREM2;LILRB3 |
| Fc gamma R-mediated phagocytosis | 7/91 | 1.6x10^-8^ | 3.9x10^-7^ | VASP;FCGR3A;SCIN;FCGR2A;PTPRC;ARPC1B;INPP5D |
| Th17 cell differentiation | 7/107 | 5.0x10^-8^ | 9.7x10^-7^ | NFKBIA;HLA-DMA;CD4;HLA-DMB;NFATC2;HLA-DRA;RUNX1 |
| B cell receptor signaling pathway | 6/71 | 1.0x10^-7^ | 1.7x10^-6^ | NFKBIA;INPP5D;BLNK;NFATC2;PTPN6;LILRB3 |
| Systemic lupus erythematosus | 7/133 | 2.2x10^-7^ | 3.1x10^-6^ | CD86;C4B;FCGR3A;HLA-DMA;HLA-DMB;FCGR2A;HLA-DRA |
| Rheumatoid arthritis | 6/91 | 4.6x10^-7^ | 5.2x10^-6^ | CD86;HLA-DMA;HLA-DMB;IL18;HLA-DRA;TLR2 |
| Th1 and Th2 cell differentiation | 6/92 | 4.9x10^-7^ | 5.2x10^-6^ | NFKBIA;HLA-DMA;CD4;HLA-DMB;NFATC2;HLA-DRA |

**Supplementary Table 9.** Significant Hallmark pathways enriched for AD risk genes in the light green network

| Pathway | Overlap | P-value | Adjusted P-value | Genes |
| --- | --- | --- | --- | --- |
| Allograft Rejection | 12/200 | 1.5x10^-12^ | 3.3x10^-11^ | CD86;HLA-DMA;CD4;SPI1;HLA-DMB;PTPRC;IL18;LY86;HCLS1;HLA-DRA;PTPN6;TLR2 |
| Interferon Gamma Response | 4/200 | 3.9x10^-3^ | 0.02 | CD86;NFKBIA;HLA-DMA;PTPN6 |
| Apical Junction | 4/200 | 3.9x10^-3^ | 0.02 | CD86;CNN2;VASP;PTPRC |
| Inflammatory Response | 4/200 | 3.9x10^-3^ | 0.02 | NFKBIA;C5AR1;IL18;TLR2 |

**Supplementary Table 10.** Association of expression of light green subnetwork genes with measures of vascular damage-related proteins

| **Gene** |  | **ICAM-1** | |  | **SAA** | |  | **VCAM-1** | |
| --- | --- | --- | --- | --- | --- | --- | --- | --- | --- |
|  |  | **β** | **P-value** |  | **β** | **P-value** |  | **β** | **P-value** |
| *BLNK* |  | 0.08 | 0.19 |  | 0.18 | 0.01 |  | 0.03 | 0.67 |
| *C4B* |  | 0.22 | **1.3x10^-5^** |  | 0.21 | **1.6x10^-4^** |  | 0.26 | **7.0x10^-6^** |
| *CD4* |  | 0.10 | 0.31 |  | 0.30 | 5.9x10^-3^ |  | 0.07 | 0.53 |
| *CD86* |  | 0.18 | 6.7x10^-3^ |  | 0.15 | 0.04 |  | 0.13 | 0.07 |
| *FCGR2A* |  | 0.11 | 0.06 |  | 0.20 | 2.7x10^-3^ |  | 0.09 | 0.20 |
| *FCGR3A* |  | 0.10 | 0.04 |  | 0.17 | 7.6x10^-4^ |  | 0.13 | 0.01 |
| *HLA-DMB* |  | 0.15 | 0.01 |  | 0.16 | 0.02 |  | 0.18 | 7.0x10^-3^ |
| *HLA-DRA* |  | 0.12 | 0.04 |  | 0.14 | 0.03 |  | 0.15 | 0.02 |
| *IL18* |  | 0.15 | 0.02 |  | 0.17 | 0.01 |  | 0.08 | 0.23 |
| *INPP5D* |  | 0.31 | 1.7x10^-3^ |  | 0.36 | 8.3x10^-4^ |  | 0.23 | 0.04 |
| *NFATC2* |  | 0.28 | 0.02 |  | 0.43 | 8.1x10^-4^ |  | 0.04 | 0.77 |
| *NFKBIA* |  | 0.32 | 2.7x10^-3^ |  | 0.38 | 1.3x10^-3^ |  | 0.14 | 0.27 |
| *PTPN6* |  | 0.25 | 4.6x10^-3^ |  | 0.29 | 2.7x10^-3^ |  | 0.34 | 6.5x10^-4^ |
| *PTPRC* |  | 0.14 | 0.06 |  | 0.22 | 6.2x10^-3^ |  | 0.06 | 0.48 |
| *RUNX1* |  | 0.14 | 0.07 |  | 0.29 | 6.5x10^-4^ |  | 0.17 | 0.05 |
| *TLR2* |  | 0.16 | 8.6x10^-3^ |  | 0.22 | 1.2x10^-3^ |  | 0.16 | 0.02 |
| *VASP* |  | 0.44 | **3.7x10^-4^** |  | 0.52 | **1.0x10^-4^** |  | 0.12 | 0.40 |

ICAM-1: intercellular adhesion molecule 1; SAA: serum amyloid alpha; VCAM-1: vascular cell adhesion molecule 1. Genes from Figure 3c and Figure 3d. Results in bold surpass the multiple-testing threshold (P<4.67x10^-4^).
